# Effectiveness of Mobile Emitter Location by Cooperative Swarm of Unmanned Aerial Vehicles in Various Environmental Conditions

**DOI:** 10.3390/s20092575

**Published:** 2020-05-01

**Authors:** Jan M. Kelner, Cezary Ziółkowski

**Affiliations:** Institute of Communications Systems, Faculty of Electronics, Military University of Technology, Gen. Sylwester Kaliski Str. No. 2, 00-908 Warsaw, Poland; cezary.ziolkowski@wat.edu.pl

**Keywords:** mobile emitter localization, Doppler effect, signal Doppler frequency (SDF), unmanned aerial vehicle (UAV), swarm, wireless sensor network (WSN), urban area, line-of-sight (LOS) and non-line-of-sight (NLOS) conditions

## Abstract

This paper focused on assessing the effectiveness of the signal Doppler frequency (SDF) method to locate a mobile emitter using a swarm of unmanned aerial vehicles (UAVs). Based on simulation results, we showed the impact of various factors such as the number of UAVs, the movement parameters of the emitter and the sensors on location effectiveness. The study results also showed the dependence of the accuracy and continuity of the emitter coordinate estimation on the type of propagation environment, which was determined by line-of-sight (LOS) or non-LOS (NLOS) conditions. The applied research methodology allowed the selection of parameters of the analyzed location system that would minimize the error and maximize the monitoring time of the emitter position.

## 1. Introduction

A localization of radio wave sources plays an important role not only in military applications such as electronic warfare [1,2], but also in navigation systems [3,4], internal security [5] and search and rescue missions [6,7]. Most of the methods analyzed in the literature are applicable to static emission sources. A perpetual change of emitter position significantly hinders the implementation of location procedures. The development of unmanned aerial vehicles (UAVs), cellular [8,9], mobile ad-hoc (MANETs) and wireless sensor networks (WSNs) contributed to developing new techniques for locating mobile objects. Mobility and the lack of spatial restrictions in the UAV missions is a special property that determines the use of these vehicles both to create dynamically changing network structures and the implementation of additional communications services such as the location of emission sources. The prospects for using the UAVs to improve the quality and scope extension of communication services in fifth-generation (5G) mobile networks and in the localization systems of fixed emission sources are presented in [10,11] and [12], respectively.

Time and frequency differences of arrival (TDOAs and FDOAs) measurements performed by many sensors are some of the more commonly used techniques for estimating the position of mobile emitters [12,13,14,15]. Target tracking techniques based on the TDOA measurements in a WSN are described, i.a., in [16,17]. Sathyan et al. [16] additionally proposed the use of an extended Kalman filter (EKF). A similar approach, but for correlated TDOA and using a Gaussian mixture (GM), was proposed by Kim et al. [18].

The mentioned EKF is widely used in radar technology [19] or target tracking applications in WSNs [20]. Pathirana et al. [21] used it for a received signal strength (RSS) to estimate the position and speed of moving MANET nodes. Schmidhammer et al. [22] presented a technique for tracking mobile scatterers as secondary radio emission sources. This approach was based on the delay estimation with the EKF and posterior Cramér–Rao lower bound. The EKF also allows to reduce the location error of the other methods, including a time of arrival (TOA) [23], direction of arrival (DOA) [24] and TDOA–DOA [25,26].

DOA-based methods also use the GM, interacting multiple models [24], electronic beam steering [27] and an algorithm based on the maximum entropy fuzzy clustering in a cluttered environment [28]. The algorithms of maximum entropy clustering and particle swarm optimization were used by Parvin et al. [29] for the energy-efficient tracking of targets in WSNs. Other examples of the positioning of mobile emitters were presented in a survey of location methods with a mobile receiver [30]. Furthermore, in [31], Jing et al. presented other non-cooperative target-tracking algorithms.

The location procedures outlined above, except [23], were tested for the location of mobile emitters in an open area, free space, or line-of-sight (LOS) conditions. Tracking a target moving in an urbanized area requires more complex algorithms. This is related to multipath propagation, which results from the presence of numerous terrain obstacles, especially buildings, vehicles and other urban infrastructure. Hence, in urbanized environments, non-LOS (NLOS) or mixed LOS/NLOS conditions often occur. Therefore, in such propagation environments, the estimation of received signal parameters that are the basis of the analyzed method are constricted and burdened with a significant error.

Localization techniques for this type of propagation environment are primarily based on the prediction and identification of LOS/NLOS conditions [32], followed by the application of appropriate correction, e.g., using the EKF [33]. The detection of LOS or NLOS conditions may be carried out using techniques of estimating a Rician K-factor [34,35] or an energy detector used in cognitive radio networks to assess channel occupancy [36,37]. From the viewpoint of location accuracy, the most crucial aspect is the correction mechanism used due to propagation conditions. It should be emphasized that the majority of solutions available in the literature for NLOS conditions are based on the theoretical error distributions of measured parameters, e.g., [38,39], instead of using more realistic methods of channel modeling. In the case of mobile sensors and emitters, the time-variant channel models should be used, e.g., [40,41]. In [32,42], this type of channel modeling was applied in relation to a signal Doppler frequency (SDF) method.

Some of the location methods assume that LOS conditions occur for part of sensors, whereas NLOS conditions occur for others, e.g., [23,43]. In these cases, the emitter position estimation procedure can only be based on sensors under LOS conditions. This approach, although idealistic, provides a much higher accuracy of location in urbanized environments. The solution proposed in this paper was also based on this assumption and was an extension of the idea presented in [32]. The sensors were located on UAVs moving above the urbanized area to make the mixed LOS/NLOS scenario realistic. Therefore, LOS/NLOS conditions changed randomly for each sensor. To model the probabilities of the LOS conditions depending on a UAV elevation and the type of urbanized environment, we used the distributions presented in [44]. The empirical results presented in [45] were the basis for modeling the radio channel between the emitter and sensors. We wanted to highlight that the use of the UAV swarm was one of the trends in the development of location procedures, e.g., [15,46,47,48].

Our paper was devoted to the location of mobile emission sources in an urban environment using the UAV swarm. Here, we focused our attention on assessing the effectiveness of the position estimation of an emitter moving in conditions occurring in a real propagation environment. The emitter localization considering the combined analysis of the UAV swarm, real changes in the propagation conditions and the Doppler effect, was an innovative contribution of our paper. In the proposed solution, the emitter location is implemented by the swarm of the sensors, in which an SDF procedure is performed [49,50]. This procedure was based on the analytical description of the Doppler frequency shift (DFS), which expresses the relationship between an instantaneous frequency of a received signal, the coordinates of the emission source and the sensor [51]. The SDF is the only closed-form algorithm of a frequency of arrival (FOA) [52]. In addition, the SDF and FOA belong to a narrow group of methods requiring a single sensor to locate the emission source. The SDF approach allows for the locating of the emitter based on only two current DFS measurements. The solution presented in [32] showed that the UAV swarm application provided for increasing the location accuracy of the immobile emitter. In this case, the transmitter location procedure was based on the signals received under both the LOS and NLOS conditions. In this paper, we proposed the location of the mobile emitter based on the signals received only under LOS conditions that have a random and limited occurrence range. Hence, studying the impact of the environment, emitter speed and the number of sensors in the swarm was important to assess the location efficiency. This evaluation was based on simulation tests. To solve the analyzed problem, we presented a significant SDF extension that allowed for the location of any velocity vectors of the sensor and emitter. In this paper, the proposed solution and the method of modeling mixed LOS/NLOS conditions showed its innovation and originality in relation to others presented in the literature.

The remainder of the paper is organized as follows. Firstly, the descriptions of the SDF method and simulation test procedures are provided in Section 2. Section 3 contains the assumptions, test scenario description and simulation results. A summary and final remarks are in Section 4.

## 2. SDF Method and Simulation Study Procedure

### 2.1. SDF Method in Location

The SDF method was based on the analytical description of the relationship between the coordinates of the emitter, $x_{e}\left(t\right)=\left(x_{e}\left(t\right),y_{e}\left(t\right),z_{e}\left(t\right)\right),$ and receiver, $x_{s}\left(t\right)=\left(x_{s}\left(t\right),y_{s}\left(t\right),z_{s}\left(t\right)\right),$ and the DFS, $f_{D}\left(t\right)=f_{D}\left(x_{e},x_{s},t\right),$ of the received signal [52]:

(1) $$f_{D}\left(t\right)=\frac{f_{c}}{c}\frac{v_{x}\left(t\right)\left(x_{e}\left(t\right)-x_{s}\left(t\right)\right)+v_{y}\left(t\right)\left(y_{e}\left(t\right)-y_{s}\left(t\right)\right)+v_{z}\left(t\right)\left(z_{e}\left(t\right)-z_{s}\left(t\right)\right)}{\sqrt{{\left(x_{e}\left(t\right)-x_{s}\left(t\right)\right)}^{2}+{\left(y_{e}\left(t\right)-y_{s}\left(t\right)\right)}^{2}+{\left(z_{e}\left(t\right)-z_{s}\left(t\right)\right)}^{2}}},$$

where $f_{c}$ is a carrier frequency of the transmitted signal, $v\left(t\right)=v_{s}\left(t\right)+v_{e}\left(t\right)=\left[v_{x}\left(t\right),v_{y}\left(t\right),v_{z}\left(t\right)\right]$ is a resultant velocity vector of the transmitter, $v_{e}\left(t\right),$ and receiver, $v_{s}\left(t\right),$ while c means the speed of light.

If the transmitter is static, $v_{e}\left(t\right)=0,$ and the receiver moves towards the OX axis at a constant speed, $v_{s}\left(t\right)=\left[v_{x},0,0\right]=\mathrm{const}.$ and $x_{s}\left(t\right)=\left(x_{s}\left(t\right),y_{s}\left(0\right),z_{s}\left(0\right)\right),$ then Equation (1) takes the form [51]:

(2) $$f_{D}\left(t\right)=\frac{f_{c}}{c}\frac{v_{x}\left(x_{e}-x_{s}\left(t\right)\right)}{\sqrt{{\left(x_{e}-x_{s}\left(t\right)\right)}^{2}+{\left(y_{e}-y_{s}\right)}^{2}+{\left(z_{e}-z_{s}\right)}^{2}}}.$$

If the DFS measurements are made in two interception intervals, $t_{1}$ and $t_{2},$ we can estimate the coordinates of the emission source by transforming Equation (2). The final formulas are as follows [49,50]:

(3) $${\tilde{x}}_{e}=x_{s}\left(0\right)+v_{x}\frac{t_{1}p\left(t_{1}\right)-t_{2}p\left(t_{2}\right)}{p\left(t_{1}\right)-p\left(t_{2}\right)},$$

(4) $${\tilde{y}}_{e}=y_{s}\left(0\right)\pm \sqrt{{\left(v_{x}\frac{\left(t_{2}-t_{1}\right)p\left(t_{1}\right)p\left(t_{2}\right)}{p\left(t_{1}\right)-p\left(t_{2}\right)}\right)}^{2}-{\left({\tilde{z}}_{e}-z_{s}\left(0\right)\right)}^{2}},$$

where $p\left(t\right)=\sqrt{{\left[\left(f_{c}v_{x}\right)/\left(c{\tilde{f}}_{D}\left(t\right)\right)\right]}^{2}-1}$ and ${\tilde{f}}_{D}\left(t\right)$ is the estimated DFS.

For an assumption that ${\tilde{z}}_{e}=z_{e}$ is known, Equations (3) and (4) describe the coordinates of the localized object on the OXY plane. This assumption was the basis for the research scenarios analyzed in the remainder of the paper. In the SDF, determining the three coordinates of the emitter position required changing the direction of the receiver movement [53].

The method, which was based on Equations (3) and (4), had numerous limitations related to the stability of the receiver speed and the lack of transmitter mobility. Therefore, in Section 2.6, we presented the novel SDF extension that allowed considering the variable movement of the sensor and emitter in any direction on the OXY plane.

Equations (3) and (4) are used to estimate the object coordinates only under LOS conditions. In the case of a real propagation environment, the range of LOS areas is limited and random. Under these conditions, the use of the UAV swarm, whose trajectories are varied, allows for obtaining the relatively continuous monitoring of the emitter position. Additionally, the use of several sensors placed on UAVs provided the opportunity to resolve an ambiguity of Equation (4) and minimize the error associated with the adopted assumption, i.e., that ${\tilde{z}}_{e}=z_{e}$ is known. 

### 2.2. Diagram of Simulation Procedure

**Figure 1:**
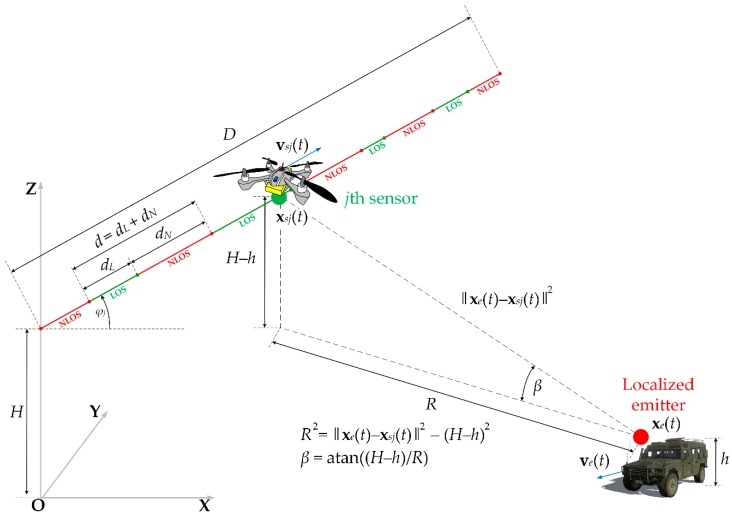
Spatial geometry of the simulation scenario for the selected sensor.

**Figure 2:**
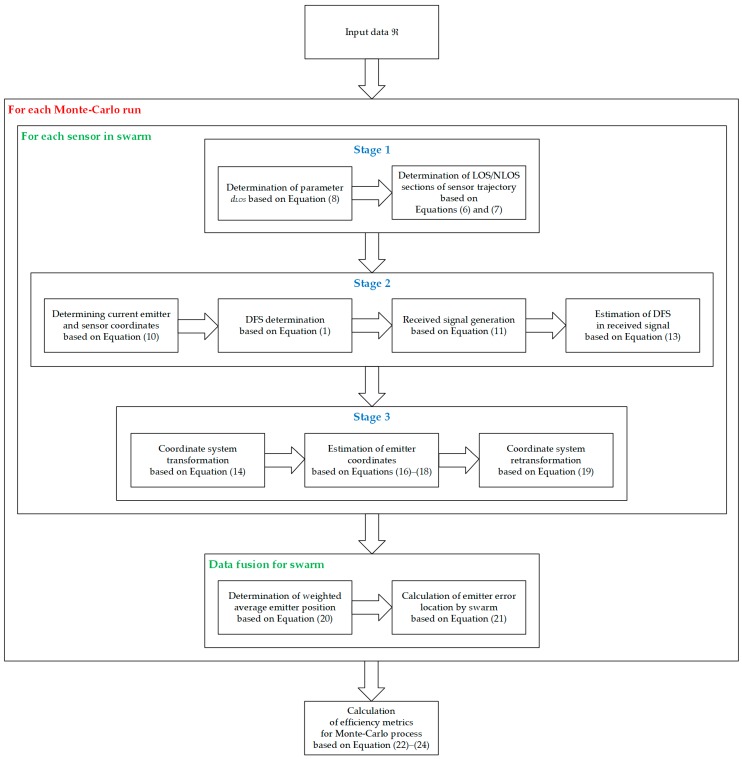
Generalized diagram of the simulation procedure.

The effectiveness evaluation of the analyzed methodology for monitoring the moving emitter position was based on the results of the simulation tests. The purpose of this research was to determine the location accuracy and monitoring continuity as a function of the number of sensors, type of propagation environment and the speed of the monitored object. Figure 1 illustrates a spatial geometry of the considered study scenario for a single sensor (free clipart of a Humvee is from [54]), while a generalized diagram of the simulation procedure is depicted in Figure 2.

The simulation studies were carried out as a Monte-Carlo process. In this case, for the input data, in each Monte-Carlo run, a comprehensive process of locating the mobile emitter by the swarm was carried out. The simulation tests for each sensor consisted of three stages:

- generation of the sensor trajectory division into sections with LOS and NLOS conditions;
- on the route sections with LOS conditions, the DFS estimation for the resultant velocity vector for the analyzed sensor and the mobile emitter;
- estimating the current emitter position relative to the vector of the sensor.

These steps were followed by a data fusion in the reference sensor. Based on the results obtained in each Monte-Carlo run, the metrics to assess the effectiveness of the emitter monitoring were determined.

### 2.3. Input Data for Simulation Studies

The primary input data for the simulation tests were a set ℜ of following parameters and characteristics:

- the coordinates of the initial position of each sensor, $x_{s}\left(0\right)=\left(x_{s}\left(0\right),y_{s}\left(0\right),z_{s}\left(0\right)\right),$ and monitored emitter, $x_{e}\left(0\right)=\left(x_{e}\left(0\right),y_{e}\left(0\right),z_{e}\left(0\right)\right);$
- the velocity vectors of individual sensors, $v_{s}\left(t\right)=\left[v_{sx}\left(t\right),v_{sy}\left(t\right),v_{sz}\left(t\right)\right],$ and the emitter, $v_{e}\left(t\right)=\left[v_{ex}\left(t\right),v_{ey}\left(t\right),v_{ez}\left(t\right)\right];$
- the probability $P_{LOS}\left(Env,\beta \right)$ of the LOS conditions occurring on the sensor trajectory as a function of the type of propagation environment Env and the elevation angle β of the sensor relative to the emitter position (see Figure 1);
- the carrier frequency $f_{c},$ an emission type and the bandwidth B of the transmitted signal;
- processing parameters of the received signals such as a sampling rate $f_{s}$ and minimum acquisition time Δt conditioning the determination of a single DFS.

### 2.4. Stage 1. Generation of LOS/NLOS Sections on Sensor Trajectory

The goal of the first stage was to divide the trajectories of individual sensors into two state sections of LOS and NLOS. We used the Poisson process to model the random occurrence of a LOS state. This meant that the lengths of the trajectory sections, which were determined by the subsequent moments of the appearance of the LOS state, are described by an exponential distribution:

(5) $$f\left(d\right)=d_{f}\exp\left(-\frac{d}{d_{f}}\right),$$

where $d_{f}$ is the average length of the trajectory section determined by successive moments of the LOS conditions, $d=d_{L}+d_{N}$ (see Figure 1) and $d_{L}$ and $d_{N}$ are the trajectory sections of the sensor, where LOS and NLOS conditions occur, respectively.

An analogous distribution describes the length $d_{L}$ of sections with LOS conditions, viz.

(6) $$f\left(d_{L}\right)=d_{LOS}\exp\left(-\frac{d_{L}}{d_{LOS}}\right),$$

where $d_{LOS}$ is the average length of the trajectory section where LOS conditions occur.

To use the above distributions to generate the trajectory division of each sensor into the LOS, $\left\{d_{L}\right\},$ and NLOS, $\left\{d_{N}\right\},$ sections, knowledge of $d_{f}$ and $d_{LOS}$ is necessary. Based on the Erlang B formula for a single sensor trajectory:

(7) $$P_{LOS}\left(Env,\beta \right)=\frac{\frac{d_{LOS}}{d_{f}}}{1+\frac{d_{LOS}}{d_{f}}},$$

thus:

(8) $$\frac{d_{LOS}}{d_{f}}=\frac{P_{LOS}\left(Env,\beta \right)}{1-P_{LOS}\left(Env,\beta \right)}.$$

where Env={suburban, urban, dense urban, highrise urban} [44].

Equation (8) is the basis for determining $d_{LOS}$ as a function of $d_{f},$ Env, and β, which are associated with $P_{LOS}\left(Env,\beta \right).$ For each sensor in the swarm, the division of the trajectory D into the sections of random length $\left\{d_{L}\right\}$ and $\left\{d_{N}\right\}$ is generated for the same parameters of the distributions (5) and (6), considering Equation (8) and β determined individually for the analyzed sensor. In our research, Figure 2 in [44] was the basis for determining $P_{LOS}\left(Env,\beta \right)$ for the various propagation environments.

### 2.5. Stage 2. Estimation of Doppler Frequency Shift in Received Signal

In the first step of the second stage, the resultant velocity vector of the sensor and mobile emitter is designated as

(9) $$v\left(t\right)=v_{sj}\left(t\right)+v_{e}\left(t\right)=\left[v_{x}\left(t\right),v_{y}\left(t\right),v_{z}\left(t\right)\right]=\left[v_{xsj}\left(t\right)+v_{xe}\left(t\right),v_{ysj}\left(t\right)+v_{ye}\left(t\right),v_{zsj}\left(t\right)+v_{ze}\left(t\right)\right].$$

Then, based on the given velocity vectors and initial positions, subsequent emitter and individual sensor positions were determined according to the relationships:

(10) $$\{\begin{array}{l} x_{e,sj}\left(t\right)=x_{e,sj}\left(0\right)+\int_{0}^{t}v_{xe,xsj}\left(t\right)dt,\\ y_{e,sj}\left(t\right)=y_{e,sj}\left(0\right)+\int_{0}^{t}v_{ye,ysj}\left(t\right)dt,\\ z_{e,sj}\left(t\right)=z_{e,sj}\left(0\right)+\int_{0}^{t}v_{ze,zsj}\left(t\right)dt. \end{array}$$

Based on Equations (9) and (10), we determined the actual DFS according to Equation (1). The obtained values of $f_{D}\left(t\right)$ were the basis for the generation of the received baseband signal. For *j*th sensor, the signal is:

(11) $$s_{j}\left(t,\tau \right)=h_{j}\left(t,\tau \right)*a\left(\tau \right)\exp\left(2\pi if_{D_{j}}\left(t\right)\tau \right)+n_{j}\left(\tau \right),$$

where $h_{j}\left(t,\tau \right)$ and $n_{j}\left(\tau \right)$ represent a channel impulse response (CIR) and an baseband additive white gaussian noise (AWGN) for the channel of the *j*th sensor, respectively, and a(τ) is a modulating function.

Considering the propagation conditions for the UAVs, the CIRs were generated according to the methodology presented in [45].

A power spectrum density (PDS) of this signal:

(12) $$s_{j}\left(t,\tau \right)=h_{j}\left(t,\tau \right)*a\left(\tau \right)\exp\left(2\pi if_{D_{j}}\left(t\right)\tau \right)+n_{j}\left(\tau \right),$$

is the basis for estimating the DFS as an argument for which the PDS takes the maximum value:

(13) $${\tilde{f}}_{Dj}\left(t\right):S_{j}\left(t,{\tilde{f}}_{Dj}\right)=\underset{f}{\max}S_{j}\left(t,f\right).$$

### 2.6. Stage 3. Estimation of Emitter Position by Individual Sensors

**Figure 3:**
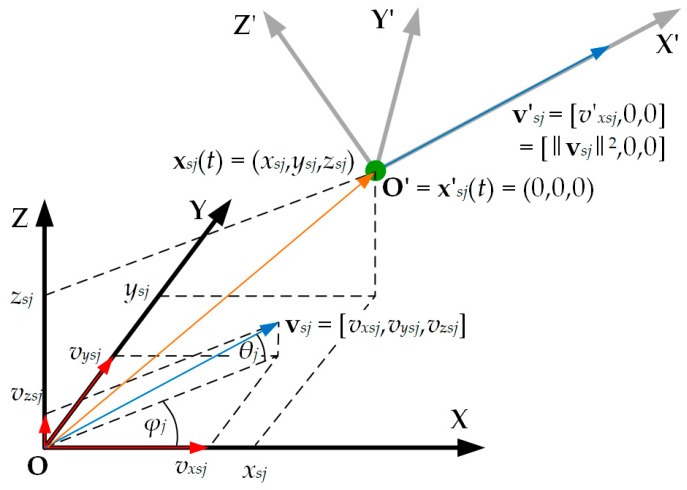
Transformation of the coordinate system relative to the sensor position and the direction of its velocity vector.

In the next stage, the current position of the emitter was estimated by individual sensors. To this aim, the OXYZ coordinate system was transformed by shifting it by the vector $x_{sj}\left(t\right)$ and rotating to the O’X’Y’Z’ system, as in Figure 3. As a result of the rotation, the direction of the O’X’ axis coincided with the velocity vector $v_{sj}\left(t\right)$ of the *j*th sensor.

Rotation angles are outlined by the following formulas:

(14) $$\begin{array}{l} \varphi_{j}\left(t\right)=\mathrm{atan}\left(\frac{v_{ysj}\left(t\right)}{v_{xsj}\left(t\right)}\right),\\ \theta_{j}\left(t\right)=\mathrm{atan}\left(\frac{v_{zsj}\left(t\right)}{\sqrt{v_{xsj}^{2}\left(t\right)+v_{ysj}^{2}\left(t\right)}}\right). \end{array}$$

This transformation of the coordinate system was carried out relative to the velocity vector and the current position of the individual sensors. The velocity vector of this sensor in the new coordinate system has the form:

(15) $${v^{\prime }}_{sj}\left(t\right)=\left[{v^{\prime }}_{xsj}\left(t\right),{v^{\prime }}_{ysj}\left(t\right),{v^{\prime }}_{zsj}\left(t\right)\right]=\left[\sqrt{v_{xsj}^{2}\left(t\right)+v_{ysj}^{2}\left(t\right)+v_{zsj}^{2}\left(t\right)},0,0\right].$$

To simplify the further analysis, we assumed that the area where the emitter moved was flat. Thus, $z_{e}\left(t\right)$ was constant and equal to the height h of the transmitting antenna. In addition, if an UAV flight altitude was H≫h, then for ground targets we may assume h≅2 m, what corresponds to the average height of the antenna for a moving human and vehicle. Then, after transformation to the O’X’Y’Z’ system, this coordinate was equal to ${\tilde{z^{\prime }}}_{e}\left(t\right)=h-H≅-H.$ In this case, the current coordinates of the mobile emitter estimated by the *j*th sensor are in the form:

(16) $${\tilde{x^{\prime }}}_{ej}\left(t\right)={\bar{v^{\prime }}}_{xsj}\left(t\right)\frac{\Delta t\cdot p_{j}\left(t-\Delta t\right)}{p_{j}\left(t\right)-p_{j}\left(t-\Delta t\right)},$$

(17) $${\tilde{y^{\prime }}}_{ej}\left(t\right)=\pm \sqrt{{\left({\bar{v^{\prime }}}_{xsj}\left(t\right)\frac{\Delta t\cdot p_{j}\left(t\right)p_{j}\left(t-\Delta t\right)}{p_{j}\left(t\right)-p_{j}\left(t-\Delta t\right)}\right)}^{2}-{\left({\tilde{z^{\prime }}}_{ej}\left(t\right)\right)}^{2}},$$

(18) $${\tilde{z^{\prime }}}_{ej}\left(t\right)={\tilde{z^{\prime }}}_{e}\left(t\right)=h-H,$$

where ${\bar{v^{\prime }}}_{xsj}\left(t\right)$ is the average sensor speed between the interception intervals t−2Δt and t, and $p_{j}\left(t\right)$ is defined as $p_{j}\left(t\right)=\sqrt{{\left[\left(f_{c}{\bar{v^{\prime }}}_{xsj}\left(t\right)\right)/\left(c{\tilde{f}}_{Dj}\left(t\right)\right)\right]}^{2}-1}.$

Equations (16) and (17) were the novel extension of the current version of the SDF described by Equations (3) and (4). This extension allowed for changing the speed and direction of the sensor, as well as considering the mobility of the emission source. We wanted to highlight that the estimation of the current emitter position was carried out ‘locally’, i.e., based on two current DFSs estimated at interception intervals t−Δt and t. The use of at least two sensors moving on different trajectories provided the opportunity to uniquely determine the coordinate sign described by Equation (17).

In the next step in the procedure, the coordinates described by Equations (16)–(18) are transformed to the original OXYZ coordinate system:

(19) $${\tilde{x}}_{ej}\left(t\right)=\left[\begin{array}{c} {\tilde{x}}_{ej}\left(t\right)\\ {\tilde{y}}_{ej}\left(t\right)\\ {\tilde{z}}_{ej}\left(t\right) \end{array}\right]=\left[\begin{array}{lll} \cos\varphi_{j}\left(t\right) & -\sin\varphi_{j}\left(t\right) & 0\\ \sin\varphi_{j}\left(t\right) & \cos\varphi_{j}\left(t\right) & 0\\ 0 & 0 & 1 \end{array}\right]\left[\begin{array}{c} {\tilde{x^{\prime }}}_{ej}\left(t\right)\\ {\tilde{y^{\prime }}}_{ej}\left(t\right)\\ {\tilde{z^{\prime }}}_{ej}\left(t\right) \end{array}\right]+\left[\begin{array}{c} x_{sj}\left(t\right)\\ y_{sj}\left(t\right)\\ z_{sj}\left(t\right) \end{array}\right].$$

### 2.7. Estimation of Weighted Average Emitter Position by Swarm

Data from individual sensors were transmitted to the reference sensor. In this research, we assumed that this sensor was stationary and located at the beginning of the adopted OXYZ coordinate system. Each sensor transmitted the following data set $\left\{{\tilde{x}}_{ej}\left(t\right){,}_{c}{\bar{v^{\prime }}}_{xsj}\left(t\right),{\tilde{f}}_{Dj}\left(t\right),t\right\},$ which was the basis for determining the weighted average emitter position for the swarm:

(20) $${\tilde{x}}_{e}\left(t\right)=\frac{\sum_{j=1}^{J}w_{j}\left(t\right){\tilde{x}}_{ej}\left(t\right)}{\sum_{j=1}^{J}w_{j}\left(t\right)},$$

where $w_{j}\left(t\right)=1-\left|{\tilde{f}}_{Dj}\left(t\right)c/\left(f_{c}{\bar{v^{\prime }}}_{xsj}\left(t\right)\right)\right|$ or $w_{j}\left(t\right)=0$ for LOS and NLOS conditions, respectively, and J represents the number of mobile sensors in the swarm.

The used weighted averaging procedure was based on a similar procedure presented in [55].

### 2.8. Calculation of Efficiency Metrics for Monte-Carlo Process

According to the methodology presented above, in each implementation of the simulation, we estimated the monitored emitter coordinates based on the UAV swarm. For the ongoing effectiveness evaluation of the proposed solution, we used the instantaneous emitter location error by individual sensors, $\Delta R_{j},$ and the average location error for the entire swarm, $\Delta R_{s}.$ These errors are defined as follows:

(21) $$\Delta R_{s,j}\left(t\right)=\sqrt{{\left\|x_{e}\left(t\right)-{\tilde{x}}_{e,ej}\left(t\right)\right\|}^{2}}.$$

The use of the Monte-Carlo method in relation to this simulation procedure provided a statistical assessment of the effectiveness of the continuous monitoring of the current emitter position. We use a root-mean-square error (RMSE) to evaluate the coordinate estimation error:

(22) $$RMSE=\sqrt{\frac{1}{M}\sum_{m=1}^{M}\Delta R_{\mathrm{avg},m}}=\sqrt{\frac{1}{M}\sum_{m=1}^{M}\frac{1}{t_{LOS,m}}\int_{0}^{t_{LOS,m}}\Delta R_{s,m}\left(t\right)dt},$$

where $\Delta R_{s,m}\left(t\right)$ is the error determined based on Equation (20) in the *m*th Monte-Carlo run, $\Delta R_{\mathrm{avg},m}$ is the average value of $\Delta R_{s,m}\left(t\right)$ determined for a total time $t_{LOS,m}$ spent by the swarm (i.e., at least one of the sensors) under the LOS conditions and M is the number of the Monte-Carlo run.

In the simulation studies, we analyzed the following effectiveness measures:

- a cumulative distribution function (CDF) of the location error, $F\left(\Delta R_{s}\right),$ obtained for the Monte-Carlo process;
- an average percentage of flight-time under the LOS conditions for sensors in a swarm:
- an effectiveness factor (EF) of the emitter monitoring by a swarm:
  (23) $$\tau_{LOS}=\frac{1}{M}\sum_{m=1}^{M}\frac{t_{LOS,m}}{T}\cdot 100\%,$$
  where T is the analyzed sensor flight-time along the trajectory of length D;
  (24) $$EF=\frac{D}{RMSE}\cdot \frac{\tau_{LOS}}{100\%}.$$
  where $\tau_{LOS}$ allows for percentage-wise evaluating the emitter monitoring time by a swarm on a mission. EF is a relative measure that allows a joint assessment of both monitoring time continuity and average location error.

The application of the Monte-Carlo method in the developed simulation procedure provided a statistical effectiveness assessment of the presented method of monitoring the mobile emitter position using the UAV swarm. The accuracy and continuity of the location process was determined as a function of the number of sensors, type of propagation environment, flight altitude of the sensors and emitter speed.

## 3. Simulation Studies and Results

The simulation tests were carried out in the MATLAB environment in two stages. In the first stage, we showed examples of the emitter location using the swarm consisting of J=5 UAVs. In this case, we considered the determined position and velocity of the emitter. In the second stage, using the Monte-Carlo method, we analyzed the impact of the number of sensors in the swarm, type of urbanized environment, the sensor flight altitude, and emitter speed on the location effectiveness. In each Monte-Carlo run, the position and motion direction of the emitter and the LOS/NLOS conditions for each sensor were random.

### 3.1. Assumptions and Scenario in Simulation Tests

**Figure 4:**
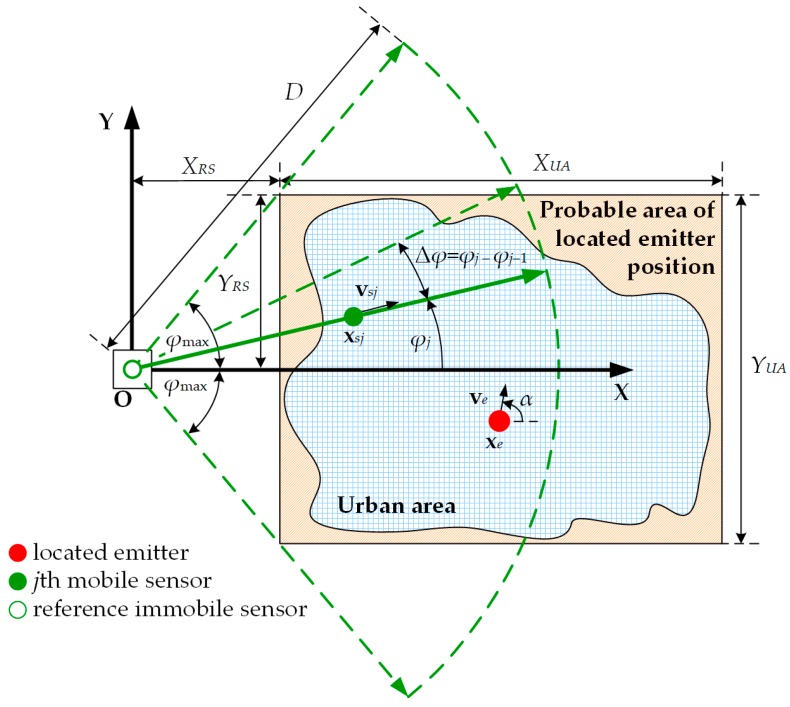
Spatial scenario of the simulation studies.

In the simulation studies, we used the spatial scenario presented in Figure 4.

The choice of simulation scenario parameters (i.e., swarm size, propagation conditions, emitter speed, and UAV flight altitude) was closely related to the SDF procedure because the emitter position relative to the UAV motion trajectory determined the accuracy of its locating. Therefore, in the simulation studies, we used the widest possible diversity of the UAV trajectory directions relative to the area of occurrence of the localized object. In this scenario, we assumed that the monitored emitter was located in an urban area that was limited by a square with dimensions $X_{UA}\times Y_{UA}.$ To simplify the simulation procedure, the analyzed area was flat and the transmitting antenna was at the constant height. We used a uniform distribution limited to the defined urbanized area $X_{UA}\times Y_{UA}$ to obtain the random initial emitter position on the OXY plane, $x_{e}\left(0\right)=\left(x_{e}\left(0\right),y_{e}\left(0\right),z_{e}\left(0\right)\right)=\left(x_{e}\left(0\right),y_{e}\left(0\right),h\right).$ In each Monte-Carlo simulation, we assumed that the emitter speed $v_{e}$ was constant. However, the velocity direction, α, on the OXY plane was random. In this case, we also used the uniform distribution in the range 〈−180°,180°). Thus, the velocity vector may be described as

(25) $$v_{e}=\left[v_{ex},v_{ey},v_{ez}\right]=\left[v_{e}\cos\alpha ,v_{e}\sin\alpha ,0\right].$$

The swarm command post was associated with a stationary reference sensor located west of the urbanized area. The data from the mobile sensors were transmitted to the reference sensor, where the data fusion and estimation of the average emitter position were implemented based on data from the entire swarm. The reference sensor position was associated with the origin of the coordinate system. In this coordinate system, the northwestern vertex of the urban area was located at $\left(X_{RS},Y_{RS}\right).$ According to Figure 4, we adopted $Y_{RS}=Y_{UA}/2$ and $X_{RS}=Y_{RS}\tan\left(\varphi_{\max}\right).$

The *j*th mobile sensor in the swarm moved at a constant speed $v_{s}$ and direction $\varphi_{j}$. The motion directions of the sensors were determined to ensure the uniform coverage of the monitored area in the sector limited to $\langle -\varphi_{\max},\varphi_{\max}\rangle .$ This meant that the angular separation Δφ between the adjacent sensors was constant and equal to $\Delta \varphi =2\varphi_{\max}/\left(J-1\right).$ Thus, the movement direction of the *j*th sensor is determined from the formula:

(26) $$\varphi_{j}=\{\begin{array}{lll} \varphi_{\max}-2\varphi_{\max}\left(j-1\right)/\left(J-1\right)=\varphi_{\max}-\left(j-1\right)\Delta \varphi & \mathrm{for} & j=1,2,\ldots ,J>1,\\ \varphi_{\max} & \mathrm{for} & j=J=1, \end{array}$$

while the sensor velocity is defined as

(27) $$v_{sj}=\left[v_{sxj},v_{syj},v_{szj}\right]=\left[v_{s}\cos\varphi_{j},v_{s}\sin\varphi_{j},0\right].$$

Additionally, we assumed that the movement analysis of each sensor began at the same time and point located at the height H above the origin of the OXYZ coordinate system, i.e., at $x_{s}\left(0\right)=\left(x_{s}\left(0\right),y_{s}\left(0\right),z_{s}\left(0\right)\right)=\left(0,0,H\right).$ The fixed flight altitude, H, and the trajectory length, D, were the same for each sensor.

Most simulation parameters were adopted based on the literature on other location methods or ground-to-air channel characteristics, where UAVs were used, e.g., [45,56,57]. It allowed for making the simulation tests more realistic.

Other assumptions adopted in the simulation tests were as follows:

- the dimensions of the urbanized area were $X_{UA}\times Y_{UA}=3000\;m\times 3000\;m;$
- three types of propagation environments, Env, were analyzed: suburban, urban, and dense urban; to evaluate the occurrence probability of LOS/NLOS conditions for these areas, we used $d_{f}=500\;m$ as described in Section 2.4; $P_{LOS}\left(Env,\beta \right)$ for the analyzed Env and specific sensor elevation β (see Figure 1) were determined based on the distributions presented in Figure 2 of [44];
- the emitter antenna height was equal to h=2 m;
- the considered emitter speeds were $v_{e}=\left\{0,1,2,5,10\right\}m/s;$
- the emitter transmitted a differential phase-shift keying (DPSK) signal with bandwidth B=400 kHz and at the carrier frequency $f_{c}=5\;\mathrm{GHz}$ (e.g., [45,56,57]);
- an angular width of the monitored sector was $2\varphi_{\max}=90{}^{\circ}\Rightarrow \varphi_{\max}=45{}^{\circ};$
- the considered number of sensors in the swarm were 1≤J≤10;
- the trajectory length of each mobile sensor was equal D=3000 m;
- the speed of each mobile sensor was equal to $v_{s}=100\;m/s$ (e.g., [16,45,56,57]);
- the flight time along the trajectory for each sensor was $T=D/v_{s}=30\;s;$
- the considered flight altitudes for the mobile sensors were H={100,200,500} m (e.g., [45,56,57]);
- the radio channel including attenuation, CIR and Rician factor was modeled under the methodology described in [45];
- the minimum signal-to-noise ratio (SNR) for the signals received by the mobile sensors was $SNR_{\min}=3\;\mathrm{dB}$ (e.g., [58]);
- the receiver parameters used in each sensor were: $B_{s}=500\;\mathrm{kHz}$—the bandwidth of the received signal, $f_{s}=2B_{s}=1000\;\mathrm{kS}/s$—sample rate, $B_{d}=10\;\mathrm{kHz}$—a bandwidth of a decimation filter, Δf=0.05 Hz—a spectrum resolution (i.e., the basic frequency of signal analysis);
- the estimation method of the DFS in the received signal was analogous to that presented in [59];
- the signal recording time required to determine a single DFS value was equal Δt=0.1 s;
- the number of Monte-Carlo runs was M=200.

### 3.2. Sample Simulation Results for Determined Position of Emitter

In these tests, we defined additional assumptions:

- suburban or urban area;
- the initial emitter position was $x_{e}\left(0\right)=\left(2000,500,2\right)\;m;$
- the emitter velocity was defined by $v_{e}=1\;m/s$ and α=90°;
- the number of mobile sensors in the swarm was J=5;
- the flight altitude of the mobile sensors was H=500 m.

**Figure 5:**
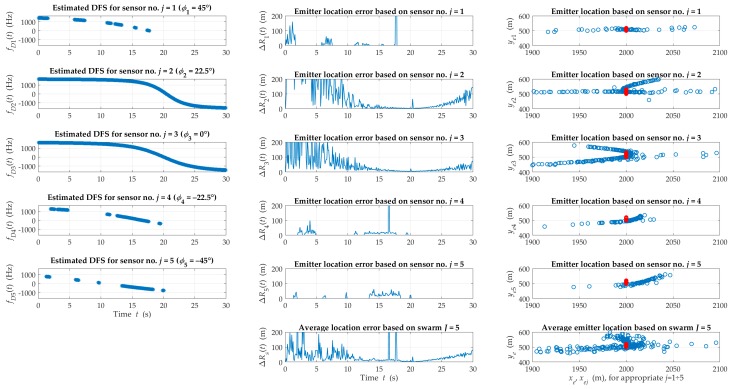
Examples of instantaneous DFS, location errors and the estimated emitter position obtained based on the individual sensors and swarm for a suburban area.

**Figure 6:**
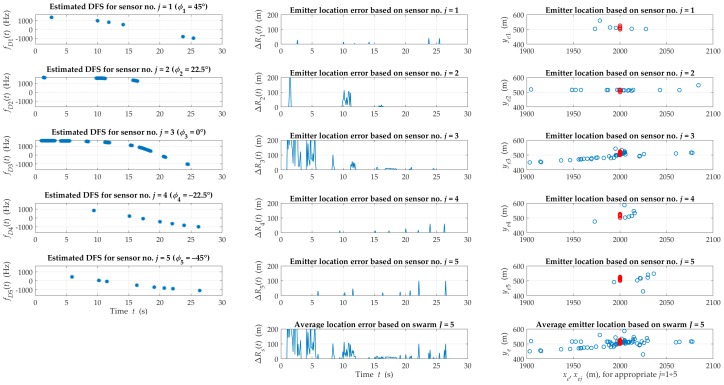
Examples of instantaneous DFS, location errors and the estimated emitter position obtained based on the individual sensors and swarm for an urban area.

Figure 5 and Figure 6 illustrate exemplary instantaneous DFSs, location errors and the estimated emitter position on the OXY plane obtained based on individual sensors and the swarm for suburban and urban terrain, respectively.

DFS graphs depict time intervals with LOS conditions that are the basis of the SDF-based localization procedure. We can notice that the occurrence probability of the LOS conditions is much higher for the suburban than the urban areas. This affects the continuous monitoring possibility of the signal source by the swarm. This probability is also related to the sensor motion direction relative to the emitter in the azimuth plane. (e.g., for sensors j=3 and j=2 were higher than for the others). If more than one sensor is under LOS conditions at an interception interval, the emitter position is weighted averaging by the swarm. In Figure 5, the graphs of the average location error for the swarm and errors for individual sensors show that the resultant error was reduced due to this averaging. The average estimation errors that were obtained for two different environments (see Figure 5 and Figure 6) assumed similar values. However, time intervals, where the emitter position was not monitored, occurred more often in the urban environment.

### 3.3. Impact of Swarm Size

**Figure 7:**
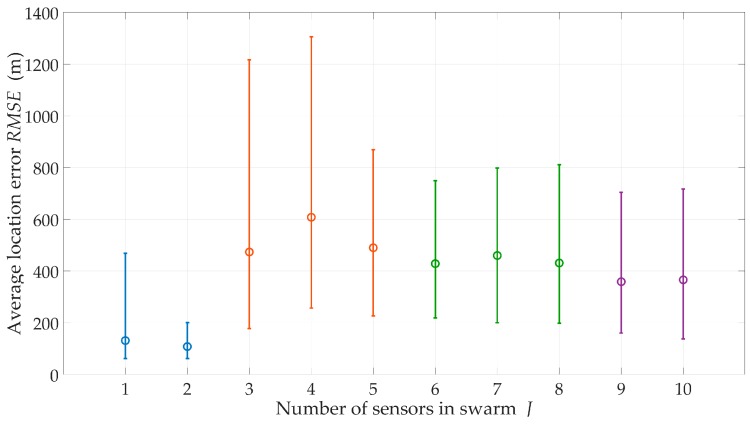
Average location error versus the number of sensors in the swarm for a suburban area.

**Figure 8:**
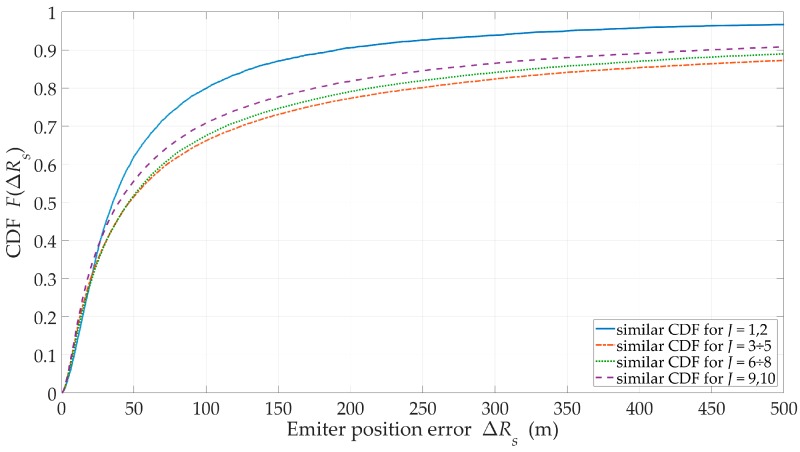
CDFs of location error versus number of sensors in swarm for suburban area.

**Figure 9:**
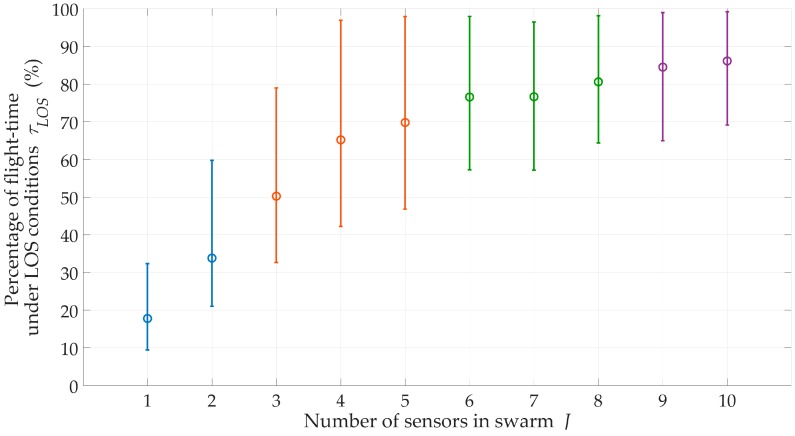
Percentage of flight-time under LOS conditions versus number of sensors in swarm for suburban area.

**Figure 10:**
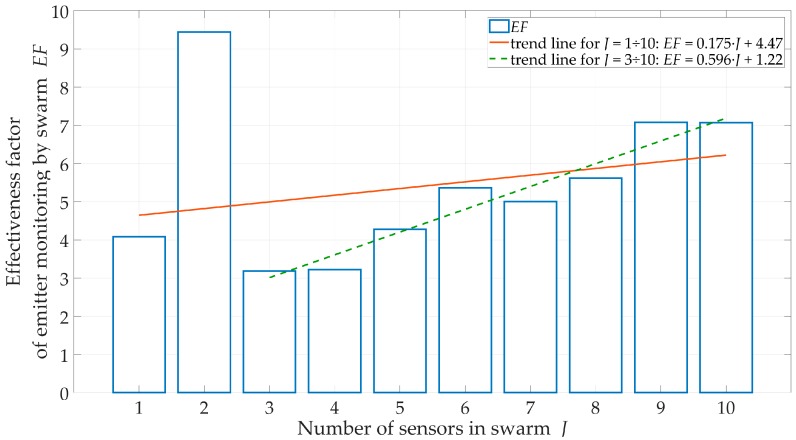
EF versus number of sensors in swarm for suburban area.

We evaluated the effect of the number J of sensors on the swarm for a suburban environment, H=200 m, and $v_{e}=1\;m/s.$ The obtained location accuracy in the form of the RMSE and CDF are shown in Figure 7 and Figure 8, respectively. Additionally, the percentage of sensor flight-time under the LOS conditions and EF are shown in Figure 9 and Figure 10, respectively.

The obtained CDFs and average errors showed that the emitter location was the most accurate for J=1 and J=2. According to Equation (26), these sensors moved in the extreme directions $\pm \varphi_{\max}$ of the monitoring sector. This location of the motion trajectory relative to the possible area of the emitter position ensured high DFS variability during the sensor movement. As a result, we obtained an increase in location accuracy. On the other hand, Figure 9 showed that in these cases, $\tau_{LOS}<35\%.$ This meant that despite the high accuracy, the localization of the mobile emitter by a single sensor could be realized only in selected time intervals. For J≥3, the accuracy of the method did not significantly depend on the number of sensors in the swarm. However, the more sensors in the swarm, the longer the monitoring time of the emitter position could be.

For suburban areas, H=500 m, and J=5, the swarm provides monitoring during 50 ÷ 95% of mission time. In Figure 10, trend lines of EF show that as the sensor number increases, the effectiveness (continuity and accuracy) of the emitter position monitoring increases.

### 3.4. Influence of Propagation Environment

**Figure 11:**
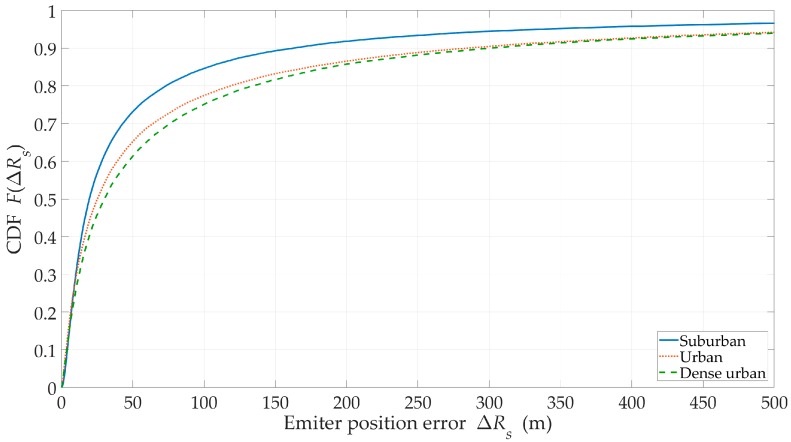
Cumulative distribution function (CDFs) of the location error versus the different types of urbanized areas.

The impact assessment of the propagation environment type is carried out for J=5, H=500 m, and $v_{e}=1\;m/s.$ In these studies, we consider three types of urbanized areas, i.e., suburban, urban, and dense urban. The obtained simulation results in the form of CDFs of location error are depicted in Figure 11, while the remaining efficiency metrics are included in Table 1.

A comparison of the results for the different environment types showed that the highest accuracy was obtained for the suburban terrains. For urban and dense urban areas, the location errors were similar. The terrain type, where the emitter is located, has a significant impact on the probability of the LOS conditions for the UAVs (see Section 2.4), which determines the value of $\tau_{LOS}$ (see Table 1). The obtained results showed that, depending on the type of propagation environment, the flight altitude of the sensors should be appropriately selected. The impact of this parameter is shown in Section 3.5. The results in Table 1 enable the effectiveness evaluation of the localization procedure for the various environmental conditions. They show that along with the worsening propagation conditions, both the location accuracy (see RMSE) and $\tau_{LOS}$ decreased. As a result, the EF, which was a measure of the location procedure effectiveness, significantly reduced. In this case, it is possible to offset this decline by increasing the number of sensors in the swarm.

### 3.5. Impact of Sensor Flight Altitude

**Figure 12:**
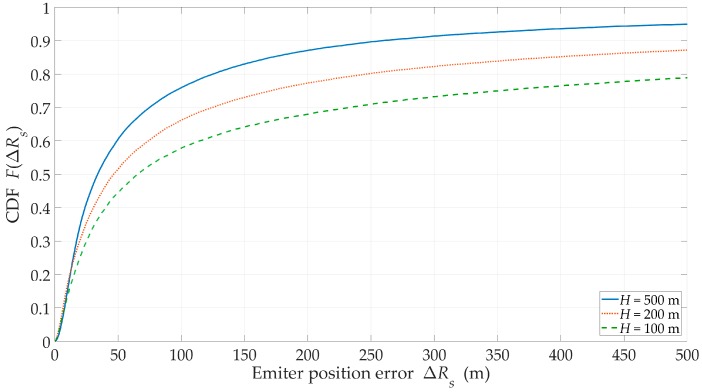
CDFs of the location error versus the different flight altitudes for a suburban area.

The effect of the UAV flight altitude was tested for suburban area, J=5, and $v_{e}=1\;m/s.$ In this study, three heights were considered, i.e., H={100,200,500} m. Figure 12 illustrates the CDFs of location error for the different sensor flight altitudes. The parameters that enabled a quantitative assessment of the monitoring process effectiveness are included in Table 2.

We can see that with the increase in the flight altitude, both the location accuracy and $\tau_{LOS}$ increased. As a result, we received over a tenfold increase in EF between H=100 m and H=500 m. This showed that we may have compensated for changes in propagation conditions related to the environment by increasing the sensor altitude.

### 3.6. Influence of Emitter Speed

**Figure 13:**
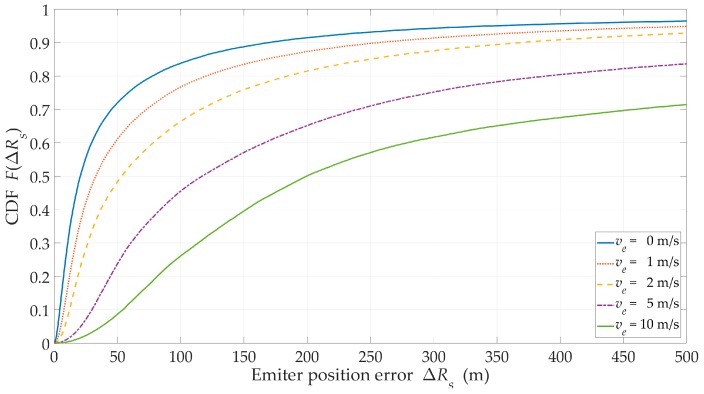
CDFs of the location error versus the emitter speed for a suburban area.

The evaluation of the impact of the emitter speed on the accuracy of the proposed approach was carried out for $v_{e}=\left\{0,1,2,5,10\right\}m/s.$ In assessing the effectiveness of most of the location methods, the location estimation of the stationary object (i.e., $v_{e}=0$) was most often considered. $v_{e}=1\;m/s$ refers to a typical pedestrian and this is a reference value in previous studies. We also analyzed the average speed of vehicles in urban areas, i.e., $v_{e}=10\;m/s=36\;\mathrm{km}/h,$ as well as the intermediate speeds. Depending on the legal regulations in a country, the speed limit in built-up areas is 30÷70 km/h. Other simulation parameters were as follows, suburban area, J=5, and H=500 m. Figure 13 shows the CDFs of the location error for the different emitter speeds. In Table 3, the values of other analyzed metrics are included.

The results presented in Table 3 show a significant relationship between the location accuracy and the emitter speed. An increase in the emitter speed from 1 m/s to 10 m/s resulted in a 15-fold decrease in the EF. This large increase in the position estimation error was caused by the approximation of the resultant sensor velocity relative to the emitter by the absolute value of the sensor velocity. This approximation associated with the velocity vector was the leading cause of the arising errors. It meant that the accuracy of the presented location procedure depended on the ratio of sensor and emitter speed. It followed that the developed procedure required introducing an additional prediction of the emitter velocity vector.

## 4. Conclusions

This paper focused on assessing the monitoring effectiveness of the mobile emitter position by the cooperative UAV swarm in various propagation conditions. We presented the novel SDF extension, which allowed for considering the variable motion of the sensors and the emitter in any direction on the OXY plane. This significantly expanded the practical use of this method. The impact analysis of the swarm size, emitter and the sensor movement parameters on the location error was performed based on the simulation results. The choice of the mentioned simulation parameters was closely related to the SDF procedure, where the spatial relationship between the emitter location and the UAV flight route influenced the positioning accuracy. In the procedure of the simulation tests, random and limited time intervals in which the mobile sensors were under LOS conditions relative to the monitored emitter were considered. The effectiveness evaluation of utilizing the UAV swarm was carried out based on the RMSE and the percentage of the occurrence time of the LOS conditions. Introducing the EF provided a measure that allowed the joint assessment of the accuracy and the continuity of the emitter position monitoring. The obtained simulation results showed that in the case of different environmental conditions and different emitter speeds, there was a differentiation in the effectiveness of the location procedure. To reduce these changes and ensure the expected monitoring parameters, the appropriate selection of flight altitude or swarm size is required. Additionally, the random way of dividing the entire sensor flight trajectory into the LOS and NLOS sections was the original solution used in the simulation procedure. This solution provides a method of determining the relationship between the environment type, flight altitude and an emitter shadowing frequency. The carried-out studies justified the need for introducing an additional procedure of the emitter speed vector prediction into the developed location method and show the possibility of obtaining the required location efficiency by selecting swarm parameters. The presented procedure of the simulation tests allows for optimizing both the selection of the location system parameters and the data processing in the SDF method. However, the impact of the additional factors related to UAV swarm management and control requires the implementation of this procedure in dedicated simulation environments such as [60,61].

## Figures and Tables

**Table 1:** Effectiveness metrics for the different types of urbanized areas.

Title 1	Propagation Environment Type, *Env*		
	Suburban	Urban	Dense Urban
*RMSE* (m)	187	280	295
$\tau_{LOS}$ (%)	96	68	43
*EF* (−)	15.4	7.2	4.4

**Table 2:** Effectiveness metrics for the different flight altitudes.

Title 1	Sensor Flight Altitude, *H*		
	100 m	200 m	500 m
*RMSE* (m)	654	518	187
$\tau_{LOS}$ (%)	31	69	96
*EF* (−)	1.4	4.0	15.4

**Table 3:** Effectiveness metrics for the different emitter speeds.

Title 1	$\mathrm{Emitter}\;\mathrm{Speed},\;v_{e}$				
	0	1 m/s	2 m/s	5 m/s	10 m/s
*RMSE* (m)	142	187	252	988	2 773
$\tau_{LOS}$ (%)	96				
*EF* (–)	20.4	15.4	11.5	2.9	1.1
